# Prenatal Exposure to Ambient PM_2.5_ and Early Childhood Growth Impairment Risk in East Africa

**DOI:** 10.3390/toxics10110705

**Published:** 2022-11-18

**Authors:** Kayan Clarke, Adriana C. Rivas, Salvatore Milletich, Tara Sabo-Attwood, Eric S. Coker

**Affiliations:** Department of Environmental and Global Health, University of Florida, Gainesville, FL 32611, USA

**Keywords:** PM_2.5_, height-for-age, stunting, East Africa

## Abstract

Height for age is an important and widely used population-level indicator of children’s health. Morbidity trends show that stunting in young children is a significant public health concern. Recent studies point to environmental factors as an understudied area of child growth failure in Africa. Data on child measurements of height-for-age and confounders were obtained from fifteen waves of the Demographic and Health Surveys (DHS) for six countries in East Africa. Monthly ambient PM_2.5_ concentration data was retrieved from the Atmospheric Composition Analysis Group (ACAG) global surface PM_2.5_ estimates and spatially integrated with DHS data. Generalized additive models with linear and logistic regression were used to estimate the exposure-response relationship between prenatal PM_2.5_ and height-for-age and stunting among children under five in East Africa (EA). Fully adjusted models showed that for each 10 µg/m^3^ increase in PM_2.5_ concentration there is a 0.069 (CI: 0.097, 0.041) standard deviation decrease in height-for-age and 9% higher odds of being stunted. Our study identified ambient PM_2.5_ as an environmental risk factor for lower height-for-age among young children in EA. This underscores the need to address emissions of harmful air pollutants in EA as adverse health effects are attributable to ambient PM_2.5_ air pollution.

## 1. Introduction

Ambient air pollution is higher in low- and middle-income countries (LMICs) compared with higher-income countries. Consequently, childhood morbidity and mortality related to prenatal ambient air pollution exposure are assumed to be disproportionately higher in LMICs [1]. However, there is minimal epidemiological data to substantiate these assumptions, particularly for the African continent.

The East African countries of Uganda, Rwanda, Ethiopia, Burundi, Kenya, and Tanzania are low-income countries [2,3] with varying ambient air pollution levels. East Africa shares a considerable burden of childhood mortality, accounting for more than half of all under-five children mortality in Sub-Saharan Africa [4]. Air pollution has been considered a major public health concern as a top risk factor for mortality in these countries and accounts for almost 1 million deaths annually. Cooking with solid fuels in households is also a significant and persistent source of air pollution in sub-Saharan Africa [5,6].

The ambient air pollutant of interest in this study is fine particulate matter with an aerodynamic diameter of 2.5 micrometers or less (PM_2.5_). Ambient PM_2.5_ is ubiquitous and can be found increasingly in all environments, especially in growing urban cities [7,8]. It is harmful to human health, with young children and elderly populations particularly vulnerable to the effects of PM_2.5_ exposure [9,10,11]. PM_2.5_ is small enough to lodge in the alveolar region of the lower respiratory system, enabling PM_2.5_ to evade the body’s innate and adaptive immune defenses [12]. This characteristic of PM_2.5_ exposure affords some particles access to the bloodstream, and recent evidence shows that it can reach the placenta during gestation [13]. Once PM_2.5_ enters, it can lead to cardiovascular effects but may also translocate to the placenta, which can affect the fetus’s health [14,15]. Several mechanisms are being explored that explain these findings, including oxidative stress, mitochondrial dysfunction, reduced telomere length, and DNA methylation [16,17,18]. Indeed, prenatal PM_2.5_ exposure has been causally linked with adverse birth outcomes such as low birth weight and growth impairment [19].

An important and widely studied early-life population health indicator for growth impairment is the height of young children. Height-for-age measurements are used to diagnose stunting in children. Children who are two standard deviations below the average height-for-age are considered stunted [20]. Height-for-age z-score charts, used to define stunting, are recommended by the World Health Organization (WHO) to assess a child’s growth and as an indicator of undernutrition [21]. Lower height-for-age related to poor nutrition is prevalent in children under five and is linked to an increased risk of chronic disease and long-term physical and cognitive defects [16,18]. Below-average height-for-age has also been linked with adverse environmental conditions, such as climate, precipitation, drought, air quality, and social vulnerability [22].

Morbidity and mortality data for East Africa show that wasting, undernutrition, and stunting in children contribute to a significant burden of childhood diseases [23]. The pooled prevalence among under-five children is estimated at 33.94% in East Africa, well above the global stunting prevalence of 22% [23]. Although stunting has been associated with malnutrition and poverty, stunting and the determinants of childhood growth are complex and multifactorial. Recent studies conducted in LMICs consider a much broader range of environmental factors that might affect stunting [16,22,24,25], highlighting the idea of the exposome to understand the combined effects that chemical, biological, and physical stressors have on human development [26,27].

For instance, data show that poor access to water, sanitation, and hygiene (WASH) infrastructure is associated with childhood stunting [28,29,30]. Certain facets of agricultural activity have also demonstrated relationships with child growth. In Indonesia, children exposed to high levels of pesticides due to involvement in agricultural activities at school age were found to be over three times more likely to experience stunting than unexposed children [31]. In rural South Africa, prenatal exposure to insecticides associated with indoor residual spraying (e.g., pyrethroids) has been associated with lower childhood growth in early life [32]. Along with these stressors, the air pollutant, PM_2.5_, is a stressor that can be composed of pesticides and other similar chemicals.

Air pollution epidemiological literature has some data linking air pollution exposures to childhood growth outcomes. In-utero exposure to ambient PM_2.5_ was significantly associated with a higher risk of stunting among children living in Bangladesh [16]. Ambient PM_2.5_ is especially of concern in households using biomass fuel for cooking, since doing so can increase indoor air pollution [33,34] and is significantly associated with childhood stunting [35,36,37]. Tobacco smoke in the home, another contributor to poor household air quality, is also correlated with significantly higher risks of childhood stunting [37].

East African countries demonstrate similar experiences of environmental risk factors related to stunting prevalence. Poor sanitary conditions have been associated with higher stunting [38,39,40,41,42]. Agricultural risk factors are critical to consider as projections indicate future changes in Africa’s climate. Despite an estimated 77% of sub-Saharan African households that use polluting cooking fuel, the associations between biomass fuel exposure and ambient outdoor air pollution and stunting in children within this context have been understudied [43].

This study aims to test the hypothesis that prenatal exposure to ambient PM_2.5_ is negatively associated with height-for-age and stunting in East African children under five. We combine existing health, sociodemographic, and environmental datasets at the individual, household, and area levels for children in six countries within the East African Community (EAC) region. We apply multilevel linear regression modeling to these data to quantify the crude and adjusted exposure-response relationship of modeled prenatal ambient PM_2.5_ exposure on height-for-age and stunting.

## 2. Materials and Methods

### 2.1. Study Area

The East African countries included in the analysis are Uganda, Rwanda, Ethiopia, Burundi, Kenya, and Tanzania. These countries’ boundaries are connected and share an anthropological and genetic history [44]. The sources of air pollution are mostly similar in these countries, as this region is experiencing rapid urbanization but still retains extensive rural populations with a heavy reliance on the agricultural sector, subsistence agriculture, and use of solid fuels for cooking [6,45,46]. Anthropogenic sources of PM_2.5_ in the region include traffic, industrial facilities, combustion of solid waste, charcoal and wood, and agrochemical usage [47,48].

### 2.2. DHS Survey Data

**Figure 1:**
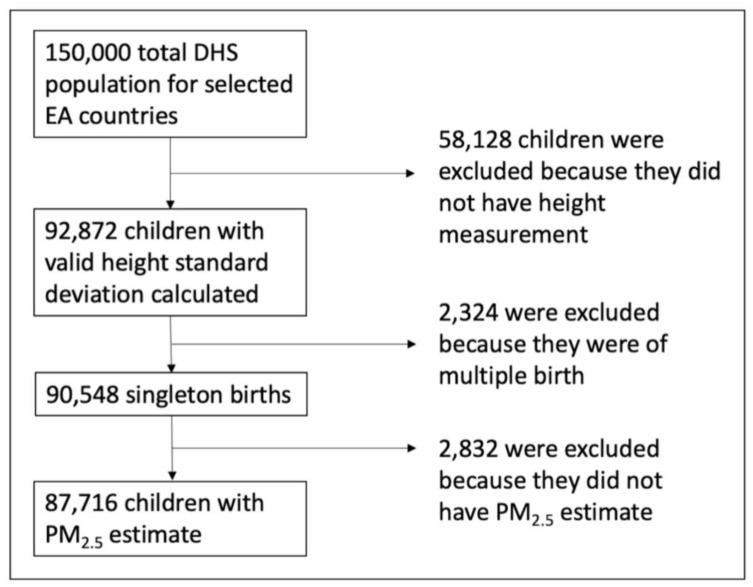
Schematic diagram of the inclusion criteria for the final sample size.

This project used nationally-representative health survey data from fifteen waves of the DHS for six countries between 2006 and 2019. Table 1 shows the waves and years of DHS data that were included for each country and the sample size of each wave based on the study’s inclusion criteria. The DHS is a two-stage cluster sample, where randomly sampled clusters of households from enumeration areas are surveyed. A detailed methodology of DHS is available elsewhere [49]. Therefore, we obtained a representative cross-section of East African children under five. The inclusion criteria for children under 5 in this study included (1) less than 60 months old at the time of the survey, (2) a valid height-for-age standard deviation value calculated, (3) singleton births, and (4) a matched PM_2.5_ estimate (see Figure 1). Other variables from the DHS were obtained for use as covariates in statistical models (see Section 2.4 Statistical Analyses).

### 2.3. Pre- and Postnatal Particulate Matter PM_2.5_ Exposure and Crop Estimation

#### 2.3.1. Predicted PM_2.5_ Surfaces

The PM_2.5_ concentration data in this research comes from the Atmospheric Composition Analysis Group (ACAG) global PM_2.5_ estimates from 1998 to 2020 [50]. ACAG provides data on ground-level prediction surfaces of average monthly PM_2.5_ concentration available at a spatial resolution of 0.1° × 0.1° (1 km × 1 km grid). ACAG derived these satellite-based global PM_2.5_ prediction surfaces by combining aerosol optical depth (AOD) data from the satellite instruments MODIS (Moderate Resolution Imaging Spectroradiometer) and MISR (Multiangle Imaging Spectroradiometer) with chemical transport models and ground-based air monitoring data [51].

#### 2.3.2. Linkage of PM_2.5_ Data with DHS Data

We spatially linked the spatial grid points of the modeled PM_2.5_ raster data with DHS data by using the latitude and longitude of each DHS cluster for each child in our study. The DHS provides these geographic coordinates with a 2 km uncertainty in urban clusters and a 5 km uncertainty in rural clusters to protect the confidentiality of participants. Therefore, we assigned each cluster a buffer zone of 2 km and 5 km for urban and rural clusters, respectively [16]. We clipped the PM_2.5_ prediction surfaces for each cluster buffer zone to assign exposures. Specifically, we used the Zonal Statistics tool in ArcGIS to calculate an average PM_2.5_ of grid-point values within each cluster buffer.

#### 2.3.3. Percent Crop Estimates

We retrieved land-use data from the European Space Agency (ESA) Climate Change Initiative (CCI) project. This project provides maps of land cover classifications at 300 m resolution from the years 1992 to 2015 (v2.0.7) and from 2016 to 2019 (v2.1.1) [52,53]. Studies have used this dataset to track land cover changes at global and regional scales [54,55]. The raster data for global land use cover for each DHS wave year was downloaded and analyzed in ArcGIS Pro. Briefly, we first clipped yearly raster data to the East Africa region. We then converted yearly crop cover raster values to polygons values by averaging the crop cover raster values for buffers of urban and rural clusters. Thus, we calculated an annual percent cropland cover for each DHS cluster, enabling a crop-cover exposure assignment for each child based on birth year.

#### 2.3.4. Prenatal and Postnatal Exposure Estimates

Prenatal PM_2.5_ exposure was estimated using the months preceding each child’s reported date of birth [16]. Specifically, for each child’s gestation period, the child’s date of birth (given by the DHS) was subtracted by 268 days, providing the starting month of gestation. Next, we calculated the child-specific PM_2.5_ prenatal exposures (including the month of birth) by averaging each month within the estimated gestation period. We calculated the child-specific postnatal exposures using the child’s date of birth and the date of the child’s height measurement (also given by DHS) [16].

### 2.4. Statistical Analyses

We calculated summary statistics for all study variables and performed bivariate analyses (ANOVA) to compare the levels of height-for-age with each covariate selected for the study. We constructed a directed acyclic graph (DAG) (see Figure S1, Supplemental Materials) to choose a candidate for testing bivariate relationships. We used general additive models (GAMs) to quantify the exposure-response relationship between prenatal PM_2.5_ exposure estimates and height-for-age, including random effects for the child’s mother, primary sampling unit (PSU) cluster, country, and month of birth. Smoothing terms were fit to adjust for the non-linear effects of birth year and percent crop coverage on height for age. Additional tested models included breastfeeding (Model 2) and postnatal PM_2.5_ exposure (Model 3) as other covariates. In addition, we used multivariable logistic regression to estimate the adjusted Odds Ratios (OR) between prenatal and postnatal ambient PM2.5 exposures for stunting. Both exposures were fit as quartiles of exposure and were included in the same model to estimate their independent effects on stunting. Since stunting is considered a common health outcome, we can assume the OR approximates the RR. Therefore, we used the adjusted ORs estimated from the model and converted them to Relative Risk (RR) of stunting using the oddsratio_to_riskratio function in the effectsize package (version 0.8.2) in R [56,57]. Using these RR estimates, we also computed a population attributable fraction (PAF) that can be attributed to ambient PM_2.5_ among children exposed prenatally and postnatally. The following formula was used to compute the PAF:

pd * (RR − 1)/RR

where pd is the proportion of cases (stunted) exposed to the risk factor (in this case, the proportion of children in the highest quartile of PM_2.5_ exposure [0.25]) and RR is the relative risk of stunting associated with the exposure [58].

#### Variables for Covariate Adjustment

Household-level covariates included the type of residence (urban or rural), wealth index, and use of polluting fuel. Maternal and child-level variables, including maternal education and information on the child’s breastfeeding history, were also analyzed. The DHS survey reported the wealth index as quintiles, which we fit as covariates in their original form. We reclassified the DHS variable for polluting fuel into a binary variable as a cleaner energy source (0 = electricity, LPG, natural gas, and biogas) versus a polluting energy source (1 = kerosene, coal, lignite, charcoal, wood, straw/shrubs/grass, agricultural crop, animal dung, briquette, bottle gas, and other). Using multivariable logistic regression, we performed multiple imputation on missing values for polluting fuel use (N = 1616). We reclassified the duration of breastfeeding variable into a binary variable indicating “ever breastfed” (≥1 month) or “never breastfed”. Missing observations for breastfeeding were also imputed (N = 9387) using multivariable logistic regression. The breastfeeding variable was conceptualized as “ever” and “never” because the analysis was robust enough to identify the effect of early-childhood nutrition on the outcome.

## 3. Results

### 3.1. Summary and Descriptive Statistics

**Figure 2:**
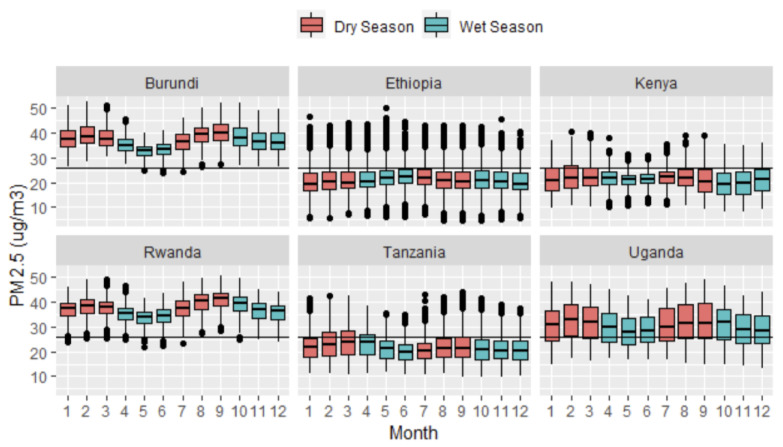
Seasonal trend of prenatal PM_2.5_ exposures stratified by child’s month of birth and by country. The horizontal black line shows the overall PM_2.5_ population average (25.83 μg/m^3^), five times above the WHO annual recommended maximum level of 5 μg/m^3^.

Our final study sample size included 87,716 children under five with prenatal PM_2.5_ exposure estimates in six East African countries (Table 2). For the complete sample, children were exposed to an entire-pregnancy average of 25.83 μg/m^3^ ambient PM_2.5_, five times above the WHO recommended level of 5 μg/m^3^ annually. Rwanda had the highest prenatal PM_2.5_ exposure on average (37.06 μg/m^3^), followed by Burundi (36.98 μg/m^3^), Uganda (30.46 μg/m^3^), Tanzania (21.81 μg/m^3^), Kenya (21.43 μg/m^3^), and Ethiopia (21.05 μg/m^3^). Generally, children born during the wet season had lower levels of prenatal PM_2.5_, and during the dry season, higher levels of prenatal PM_2.5._ We also observed higher PM_2.5_ levels in the dry seasons for Burundi, Kenya, Rwanda, Tanzania, and Uganda (Figure 2) (see Figure S2, Supplemental Materials, for annual trends). Ethiopia and Kenya had the most negligible monthly variability, with no apparent seasonal trend. Regardless of seasonal variations, children in Burundi and Rwanda experienced prenatal PM_2.5_ exposures above the mean.

**Figure 3:**
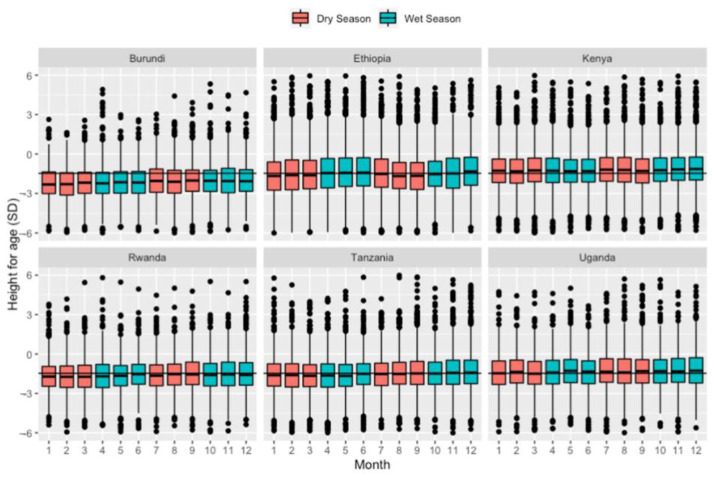
Height-for-age standard deviation by child’s month of birth for each country.

Overall, children’s height-for-age z-score in the study population was low (mean = −1.51 [95% CI: −1.49, −1.53]). The height for age Z-scores were lowest in Burundi (mean = −2.17), and the highest was in Kenya (mean = −1.19) (Table 2). Most of the country’s height-for-age distribution was around the mean for the months of birth, with little to no noticeable variation (Figure 3) (see Figure S3, Supplemental Materials, for annual trends). Height-for-age among children in Burundi was below the height-for-age mean of the study population.

The mean child age was 28.69 months, with 49.4% of the study population being comprised of girls. Most of the children in the sample resided in a rural setting (80.8%), ranging from 69.6% rural in Kenya to 91.3% rural in Burundi. The vast majority (97.5%) of the households included in the sample used solid fuel for cooking.

### 3.2. Bivariate Analyses

A bivariate analysis for each study covariate (Table 3) shows that all are associated with height-for-age at a significance level of less than 0.001, except breastfeeding (*p* > 0.05). Although breastfeeding was not significant, we included it in Model 2 because it is a known risk factor for height for age.

### 3.3. Association between Pprenatal PM_2.5_ Exposure and Height forAge

The crude model (Table 4) indicates that a 10 µg/m^3^ increase in prenatal PM_2.5_ exposure is associated with a lower height for age Z-score of −0.143 (95% CI: −0.154, −0.131). After controlling for rurality, maternal education, wealth index, percent crop cover, and use of polluting fuel (Model 1), a 10 µg/m^3^ increase of prenatal PM_2.5_ was associated with a lower height-for-age Z-score by −0.08 (−0.98, −0.62). After controlling for the child ever being breastfed (Model 2), a 10 µg/m^3^ increase of prenatal PM_2.5_ exposure was associated with a lower height-for-age by −0.095 (−0.114, −0.076). After controlling for the independent effect of postnatal PM_2.5_ exposure (Model 3), the effect of prenatal PM_2.5_ exposure on height for age was slightly attenuated (β = −0.069 [−0.097, −0.041]). The effect of postnatal PM_2.5_ exposure on height-for-age (β = −0.05 [−0.08, −0.02]) was slightly smaller than the prenatal effect estimate, but had a similar magnitude in the exposure-response relationship.

Other covariates were independently associated with height-for-age Z-score, and associations were in the expected directions (Table 4). For instance, children in rural communities had significantly lower height-for-age compared with children in urban communities. Higher maternal education, household wealth index, and ever breastfed were all independently associated with higher height-for-age Z-scores after adjustment. While using a polluting fuel was significantly associated with lower height-for-age Z-score in Model 1, this association became null after adjusting for ever breastfed (Model 2).

### 3.4. Association between Prenatal and Postnatal PM_2.5_ Exposure and Stunting

Relative to the lowest prenatal PM_2.5_ exposure group, the highest quartile of prenatal PM_2.5_ exposure is associated with a 12% (7%, 17%) higher risk of stunting (Table 5). Independent of prenatal PM_2.5_ exposure and relative to the lowest postnatal PM_2.5_ exposure group, the highest quartile of postnatal PM_2.5_ exposure is associated with an 11% (6%, 16%) higher risk of stunting. These results suggest that 2.7% and 2.5% of the population attributable risk of stunting in the study area are due to ambient prenatal PM_2.5_ and postnatal PM_2.5_ exposures, respectively. The RR of stunting for all other risk factors considered in the model are further summarized in Table 5.

## 4. Discussion

Using multiple waves of nationally representative DHS data for children under five in six East African countries, our results show a robust negative exposure-response relationship between prenatal ambient PM_2.5_ exposure and height-for-age Z-scores and a positive prenatal PM_2.5_ exposure-response relationship for stunting. Additionally, our analysis found that the overall average prenatal ambient PM_2.5_ exposure (25.83 μg/m^3^) far exceeded the WHO annual guideline for ambient PM_2.5_ (5 μg/m^3^ [59]).

From the fully adjusted models, we observe significant adverse effects on height for age in the study population (β range: −0.095 to −0.069 SD, Table 4). Our findings suggest an adverse shift in the population mean and distribution of height-for-age Z-scores due to prenatal PM_2.5_ exposure. Such population-level impacts imply that more children are likely to fall into the clinically relevant classification of stunting due to elevated PM_2.5_ levels. Indeed, the fully adjusted logistic regression model (Table 5) showed that the highest quartile of prenatal PM_2.5_ exposure is associated with a 12% higher risk of stunting compared to the lowest exposure group. The elevated risk of stunting from elevated prenatal ambient PM_2.5_ exposure underscores the clinical significance of our results.

The population attributable fraction (PAF) is a useful metric for evaluating the relative contribution that a specific risk factor has on important population health indicators. In our study, we estimated that 2.8% and 2.5% of stunting in East Africa is attributed to the highest quartiles of exposure to prenatal PM_2.5_ and postnatal PM_2.5_, respectively. The estimated PAF for stunting due to exposure to polluting cooking fuel is 7.2% in our study. Together, the highest quartiles of prenatal and postnatal PM_2.5_ exposures, along with cooking with polluting fuel, attribute to up to 12.5% of stunting risk in East Africa. This finding further highlights the need for health policy and health promotion to emphasize air pollution mitigation, for both ambient and household sources, and to achieve the global target to reduce childhood stunting.

Our results compare favorably with data from the limited number of studies that have investigated the exposure-response relationship between ambient prenatal PM_2.5_ exposure and childhood height-for-age and stunting using DHS data [16,22]. Spears et al. [16,22] found in India that a 10 μg/m^3^ increase in PM_2.5_ was associated with a 0.005 standard deviation reduction in child height [16,22]. Their effect estimate is an order of magnitude lower than what we observed in our study. This effect size difference may be explained by various study design differences, such as our use of entire-pregnancy exposure estimates or more spatially resolved exposure estimates. For example, the India study used the nearest monitoring data to reflect exposure for an entire city, which can contribute to significant exposure misclassification that would bias effects downward. Differences in population vulnerability to PM_2.5_ effects may also explain these differences.

In Bangladesh, Goyal et al. found that children prenatally exposed to levels of entire-pregnancy PM_2.5_ in the highest quartile were 1.13 times more at risk of stunting than children prenatally exposed to PM_2.5_ levels [16,22]. In our study, we observed a similar effect size, where children in the highest quartile were up to 1.12 times more at risk of stunting compared to the lowest quartile of exposure. This Bangladesh study used similar ACAG PM_2.5_ estimates. However, the ACAG estimates were only available annually at the time of their study. In contrast, our study used updated model estimates of monthly PM_2.5_ concentrations, which likely reduced exposure misclassification for our study. Moreover, we estimated postnatal exposure to control for any possible residual confounding that may be attributed to ambient PM_2.5_ exposure after the prenatal period.

Our estimates for PAF also compares favorably with recent country-level PAF estimates for ambient PM_2.5_ on risk of low birth weight (LBW) in Burundi, Ethiopia, Kenya, Rwanda, Tanzania, and Uganda [19]. Ghosh et al. (2021) estimated that a median PAF of 2.04% of LBW risk is attributable to prenatal ambient PM_2.5_ exposure in these countries, while our study estimates a prenatal ambient PM_2.5_ PAF of 2.7% for stunting [19].

Our findings are notably consistent with the existing literature concerning other child-growth risk factors. The bivariate and adjusted models showed that children residing in rural areas were likelier to have lower height-for-age and higher stunting than their urban counterparts. This is consistent with other studies identifying rurality as a risk factor for childhood growth impairment [60,61,62]. Like other studies, maternal education and wealth index was also significantly associated with better growth outcomes in our study [63,64,65]. The use of polluting fuel was significantly negatively associated with height-for-age in our study, which is also consistent with other studies that have found strong associations between childhood growth and biomass fuel for cooking [35,37].

The results on seasonal trends showed that prenatal PM_2.5_ exposure varied based on being born in the wet or dry season. Noticeably, the dry seasons had higher levels of PM_2.5_, and the wet seasons had lower levels of PM_2.5_. Previous studies also show that PM_2.5_ depends on seasonally varying factors, including weather conditions. A study conducted in Uganda found that higher monthly precipitation was significantly predictive of lower PM_2.5_ levels [66]. This finding suggests that infants born during the dry season face disproportionately higher levels of prenatal ambient PM_2.5_ exposures in our study area. However, there was no clear association between seasonality (wet versus dry) and height-for-age.

Additionally, there were differences in air pollution between countries. These differences may be partly explained by the economic development differences between countries in our study. According to the World Bank, the three countries below the population mean PM_2.5_ levels: Kenya, Tanzania, and Ethiopia, had the highest gross domestic product in 2021 [67,68].

There are several limitations to this study. There is a risk of exposure misclassification in our study attributed to maternal factors during pregnancy. These maternal factors could include moving between homes during pregnancy or gestation periods that did not go to full term. For instance, our PM_2.5_ exposure estimation assumed that the mother lived in the same house during the nine months preceding the DHS household survey, which may not be the case for some mothers. In addition, other pollutants potentially affect the outcome of height-for-age, such as sulfur dioxide, nitrogen dioxide, and ozone [69,70]. These co-pollutants were not included in the analysis as covariates because the data was unavailable for the region. Also, our study did not explore the composition of PM25. Ambient PM_2.5_ air pollution is composed of different types of chemicals, which our study could not assess.

Furthermore, we used calculated PM_2.5_ exposures with primarily satellite-driven estimates, as ground-level data was unavailable for much of the study area to validate predictions. However, the reported R^2^ from the ACAG cross-validated model was 0.90–0.92, suggesting reliable estimates [50]. When going through the inclusion criteria, several individuals were excluded (N = 63,284) from the analysis. However, we assume that these unmatched observations are missing randomly, which should not introduce selection bias in the study. Additionally, even though we used the DAG to identify the variables to be included in the models, residual confounding could still be present in the model and should be considered when interpreting the results of the estimated exposure-response relationships between prenatal and postnatal PM_2.5_.

The strengths of this study include analyzing nationally representative data for six East African countries using several waves of the Demographic Health Surveys (DHS). This approach enabled us to spatially link births reported in the DHS with monthly PM_2.5_ values and estimate PM_2.5_ prenatal exposures and effects at the individual level after controlling for multiple relevant confounding variables. Using GAMs in a hierarchical modeling framework allowed us to further account for non-linear temporal confounders, the hierarchical structure of the DHS survey itself, and height-for-age outcome. Our study also leveraged newly available high spatial and temporal resolution data on PM_2.5_ estimates provided on a global scale. While previous studies using DHS data in Bangladesh and India relied on long-term annual global PM_2.5_ values and month-of-birth PM_2.5_ measurement data, we analyzed PM_2.5_ global estimates available at a monthly scale and a prediction model that improved from previous versions. Therefore, we could derive entire-pregnancy exposure estimates incorporating between-season variability of PM_2.5_, which previous studies did not include. Therefore, our approach to exposure estimation should entail lower exposure misclassification and, thus, more refined PM_2.5_ effect estimates than the India and Bangladesh studies. Another significant strength of our analysis is the use of multiple models with different risk factors that allowed us to interrogate the robustness of prenatal PM_2.5_ effect estimates. For instance, we could control for postnatal exposures that could confound the relationship between prenatal exposures and height for age. Indeed, adjusting for postnatal PM_2.5_ resulted in attenuation in the prenatal effect estimate for height-for-age.

## 5. Conclusions

We observed a significant negative exposure-response relationship between prenatal ambient PM_2.5_ and childhood height-for-age in East Africa. We also found a positive exposure-response relationship between postnatal PM_2.5_ exposure estimates and stunting. Currently, stunting remains a high-priority issue in East Africa because it has implications for morbidity and mortality and the overall economy in the long term. These results highlight that exposure to ambient air pollution is a priority for childhood health and development, starting from gestation through early childhood. There is also a need for further expansion of ground air monitoring in these countries and substantial policy changes concerning air pollution reduction in East Africa.

## Figures and Tables

**Table 1:** Demographic Health Survey waves for each country included in the study.

Country	DHS Wave Analyzed	Sample Size
Burundi	2010	3376
	2016–2017	5876
Ethiopia	2011	8627
	2016	8319
	2019	4967
Kenya	2008–2009	4742
	2014	17,716
Rwanda	2010	3952
	2014–2015	3402
	2019	3708
Tanzania	2010	6352
	2015–2016	8722
Uganda	2006	1859
	2011	1883
	2016	4215

**Table 2:** Summary statistics of study population characteristics overall and stratified by country.

	All Countries	Burundi	Rwanda	Kenya	Tanzania	Ethiopia	Uganda
Number of children <60 months	87716	9252	11,062	22,458	15,074	21,913	7957
Mean prenatal PM_2.5_ (μg/m^3^)	25.98	37.03	37.02	22.09	23.22	19.69	31.27
Mean height-for-age z-score	−1.51	−2.17	−1.59	−1.19	−1.53	−1.54	−1.30
Percent Stunted	37.16	54.25	37.80	27.95	35.72	38.58	31.05
Mean age in months	28.69	28.72	29.09	28.99	27.66	29.12	27.94
Mean birth order	3.62	3.76	3.20	3.25	3.64	3.95	4.05
Percent Girls	49.44	49.47	49.49	49.34	50.02	49.03	49.65
Percent Rural	80.80	91.29	85.14	69.59	76.91	86.06	83.72
Percent used polluting fuel for cooking	97.48	99.85	98.99	93.41	99.37	97.53	99.63
Percent ever breastfed	98.47	99.30	99.60	98.73	98.81	96.99	98.45

Survey package functions in R were used to account for the complex stratified random sampling used by DHS.

**Table 3:** Results from bivariate analyses of each covariate’s association with height-for-age.

Covariate	Height-for-Age Mean (SD)	*p*-Value
Urban/rural status		<0.001
Urban	−1.03 (1.46)	
Rural	−1.59 (1.51)	
Maternal education		<0.001
No education	−1.68 (1.63)	
Primary	−1.51 (1.43)	
Secondary	−1.03 (1.39)	
Higher	−0.52 (1.35)	
Wealth index		<0.001
1st quintile (poorest)	−1.65 (1.60)	
2nd quintile	−1.66 (1.47)	
3rd quintile	−1.56 (1.45)	
4th quintile	−1.40 (1.44)	
5th quintile (richest)	−0.94 (1.44)	
Polluting fuel use		<0.001
Clean	−0.53 (1.36)	
Polluting	−1.49 (1.51)	
Breastfeeding status		0.2
Never	−1.53 (1.62)	
Ever	−1.47 (1.52)	

**Table 4:** Generalized additive modeling results showing crude and adjusted effect estimates for a 10 µg/m^3^ increase of prenatal PM_2.5_ exposure and height-for-age in children under 5 years old.

	Model 0	Model 1 ^a^ β (95% CI)	Model 2 ^b^ β (95% CI)	Model 3 ^c^ β (95% CI)
Prenatal PM_2.5_ exposure	−0.143 *** (−0.154, −0.131)	−0.080 *** (−0.98, −0.062)	−0.095 *** (−0.114, −0.076)	−0.069 *** (−0.097, −0.041)
Type of residence				
Urban		reference		
Rural		−0.824 *** (−1.125, −0.524)	−0.836 *** (−1.167, −0.505)	−0.955 *** (−1.287, −0.623)
Maternal education				
1st quartile		reference		
2nd quartile		0.272 * (0.031, 0.514)	0.472 *** (0.218, 0.726)	0.466 *** (0.211, 0.721)
3rd quartile		2.247 *** (1.886, 2.607)	2.602 *** (2.214, 2.990)	2.623 *** (2.232, 3.013)
4th quartile		4.707 *** (4.085, 5.329)	5.111 *** (4.420, 5.803)	5.266 *** (4.571, 5.962)
Wealth index				
1st quintile		reference		
2nd quintile		0.418 ** (0.135, 0.701)	0.366 * (0.064, 0.668)	0.466 ** (0.163, 0.770)
3rd quintile		1.464 *** (1.168, 1.761)	1.321 *** (1.013, 1.642)	1.431 *** (1.116, 1.747)
4th quintile		2.740 *** (2.430, 3.051)	2.616 *** (2.289, 2.944)	2.736 *** (2.408, 3.065)
5th quintile		5.788 *** (5.398, 6.179)	5.692 *** (5.278, 6.106)	5.775 *** (5.360, 6.191)
Use of polluting fuel				
No		reference		
Yes		−0.763 * (−1.472, −0.053)	−0.374 (−1.165, 0.418)	−0.455 (−1.250, 0.340)
Ever Breastfed				
No			reference	
Yes			0.812 * (0.002, 1.622)	0.804 ** (−0.006, 1.613)
Postnatal PM_2.5_ exposure				−0.050 *** (−0.080, −0.020)
Observations	87,716	87,716	78,329	75,949
AIC	321,509	312,053	279,426	269,255

*** *p*  <  0.01, ** *p*  <  0.05, * *p*  <  0.1. ^a^ Model 1: Smoothing terms for the year of birth, percent crop, and random effects for primary sampling unit, mother, the month of birth, and country (graph of smoothed variables can be seen in Figures S4 and S5). ^b^ Model 2: Ever breastfed added to the model. ^c^ Model 3: Postnatal PM_2.5_ exposure added to the model. CI: Confidence Interval.

**Table 5:** Adjusted relative risks of quartiles of prenatal and postnatal PM_2.5_ exposure for stunting among children under 5 years old in the East Africa Region.

	Adjusted RR (95% CI)
Prenatal PM_2.5_ exposure quartiles	
4.55–19.4 μg/m^3^	reference
20.4–24 μg/m^3^	1.00 (0.97, 1.03)
25–32.7 μg/m^3^	1.02 (0.98, 1.06)
32.7–52.4 μg/m^3^	1.12 (1.07, 1.17)
Postnatal PM_2.5_ exposure quartiles	
2.62–19.8 μg/m^3^	reference
20.8–24.1 μg/m^3^	1.02 (0.99, 1.04)
25.1–33.1 μg/m^3^	1.02 (0.98, 1.06)
34.1–74.7 μg/m^3^	1.11 (1.06, 1.16)
Type of residence	
Urban	reference
Rural	1.06 (1.03, 1.09)
Maternal education	
1st quartile	reference
2nd quartile	0.96 (0.94, 0.99)
3rd quartile	0.78 (0.74, 0.81)
4th quartile	0.58 (0.52, 0.64)
Wealth index	
1st quintile	reference
2nd quintile	0.95 (0.92, 0.97)
3rd quintile	0.88 (0.86, 0.91)
4th quintile	0.77 (0.75, 0.80)
5th quintile	0.57 (0.54, 0.60)
Use of polluting fuel	
No	reference
Yes	1.08 (0.99, 1.17)
Ever Breastfed	
No	reference
Yes	0.91 (0.84, 0.98)
Observations	75,949
AIC	94,760

The adjusted RR was computed using the modeling output of Odds Ratios estimated with multivariable logistic regression, adjusting for year of birth and percent crop as smoothing terms and random effects for primary sampling unit, mother, the month of birth, and country. Full modeling output is provided in the Supplemental Materials (see Table S1). CI: Confidence Interval; RR: Relative Risk; AIC: Akaiki information criterion.

## Data Availability

The data generated and analyzed in this study is available upon request from the corresponding author.

## Supplementary Materials

The following supporting information can be downloaded at: https://www.mdpi.com/article/10.3390/toxics10110705/s1, Figure S1: Directed Acyclic Graph; Figure S2: Trend of prenatal PM_2.5_ exposures stratified by child’s year of birth and by country; Figure S3: Trend of height-for-age stratified by child’s year of birth and by country; Table S1: Crude and adjusted odds ratios for a 10 µg/m^3^ increase of prenatal PM_2.5_ exposure and stunting in children under 5 years old. Figure S4: Plots of non-linear effects on height-for-age for (A) year of birth and (B) percent crop cover; Figure S5: Plots of non-linear effects on stunting for (A) year of birth and (B) percent crop cover.

## References

[1] World Health Organization (2018). Air Pollution and Child Health: Prescribing Clean Air.

[2] Monga C., Ogunleye E.K., Ezanin Koffi A.M., Baidoo T., Ngong V. (2019). African Development Bank Group East Africa Economic Outlook: 2019.

[3] World Bank Middle Income Countries Overview: Development News, Research, Data. https://www.worldbank.org/en/country/mic/overview.

[4] Tesema G.A., Teshale A.B., Tessema Z.T. (2021). Incidence and predictors of under-five mortality in East Africa using multilevel Weibull regression modeling. Arch. Public Health.

[5] Fisher S., Bellinger D.C., Cropper M.L., Kumar P., Binagwaho A., Koudenoukpo J.B., Park Y., Taghian G., Landrigan P.J. (2021). Air pollution and development in Africa: Impacts on health, the economy, and human capital. Lancet Planet. Health.

[6] Bauer S.E., Im U., Mezuman K., Gao C.Y. (2019). Desert dust, industrialization, and agricultural fires: Health impacts of outdoor air pollution in africa. J. Geophys. Res. Atmos..

[7] Han L., Zhou W., Li W. (2015). Increasing impact of urban fine particles (PM2.5) on areas surrounding Chinese cities. Sci. Rep..

[8] Zhang L., Wilson J.P., MacDonald B., Zhang W., Yu T. (2020). The changing PM2.5 dynamics of global megacities based on long-term remotely sensed observations. Environ. Int..

[9] US EPA EPA Health and Environmental Effects of Particulate Matter (PM). https://www.epa.gov/pm-pollution/health-and-environmental-effects-particulate-matter-pm.

[10] Wensu Z., Wen C., Fenfen Z., Wenjuan W., Li L. (2021). The Association Between Long-Term Exposure to Particulate Matter and Incidence of Hypertension Among Chinese Elderly: A Retrospective Cohort Study. Front. Cardiovasc. Med..

[11] Wang Y., Shi L., Lee M., Liu P., Di Q., Zanobetti A., Schwartz J.D. (2017). Long-term Exposure to PM2.5 and Mortality Among Older Adults in the Southeastern US. Epidemiology.

[12] Xing Y.-F., Xu Y.-H., Shi M.-H., Lian Y.-X. (2016). The impact of PM2.5 on the human respiratory system. J. Thorac. Dis..

[13] US EPA, Air Quality Planning Unit New England How Does PM Affect Human Health?. https://www3.epa.gov/region1/airquality/pm-human-health.html.

[14] D’Errico J.N., Stapleton P.A. (2019). Developmental onset of cardiovascular disease—Could the proof be in the placenta?. Microcirculation.

[15] Du Y., Xu X., Chu M., Guo Y., Wang J. (2016). Air particulate matter and cardiovascular disease: The epidemiological, biomedical and clinical evidence. J. Thorac. Dis..

[16] Goyal N., Canning D. (2017). Exposure to ambient fine particulate air pollution in utero as a risk factor for child stunting in bangladesh. Int. J. Environ. Res. Public Health.

[17] Srám R.J., Binková B., Dejmek J., Bobak M. (2005). Ambient air pollution and pregnancy outcomes: A review of the literature. Environ. Health Perspect..

[18] Sinharoy S.S., Clasen T., Martorell R. (2020). Air pollution and stunting: A missing link?. Lancet Glob. Health.

[19] Ghosh R., Causey K., Burkart K., Wozniak S., Cohen A., Brauer M. (2021). Ambient and household PM2.5 pollution and adverse perinatal outcomes: A meta-regression and analysis of attributable global burden for 204 countries and territories. PLoS Med..

[20] Lewit E.M., Kerrebrock N. (1997). Population-based growth stunting. Future Child..

[21] WHO Malnutrition in Children. https://www.who.int/data/nutrition/nlis/info/malnutrition-in-children.

[22] Spears D., Dey S., Chowdhury S., Scovronick N., Vyas S., Apte J. (2019). The association of early-life exposure to ambient PM2.5 and later-childhood height-for-age in India: An observational study. Environ. Health.

[23] Tesema G.A., Yeshaw Y., Worku M.G., Tessema Z.T., Teshale A.B. (2021). Pooled prevalence and associated factors of chronic undernutrition among under-five children in East Africa: A multilevel analysis. PLoS ONE.

[24] Vilcins D., Sly P.D., Jagals P. (2018). Environmental Risk Factors Associated with Child Stunting: A Systematic Review of the Literature. Ann. Glob. Health.

[25] Aghasili O.U. (2015). Fuel Choice, Acute Respiratory Infection and Child Growth in Uganda. Master’s Thesis.

[26] Gao P. (2021). The exposome in the era of one health. Environ. Sci. Technol..

[27] DeBord D.G., Carreón T., Lentz T.J., Middendorf P.J., Hoover M.D., Schulte P.A. (2016). Use of the “Exposome” in the Practice of Epidemiology: A Primer on -Omic Technologies. Am. J. Epidemiol..

[28] Alok K. (2010). Squatting with Dignity: Lesson from India.

[29] Hammer J., Spears D. (2013). Village Sanitation and Children’s Human Capital: Evidence from a Randomized Experiment by the Maharashtra Government. World Bank Policy Research Working Paper No. 6580.

[30] Spears D., Ghosh A., Cumming O. (2013). Open defecation and childhood stunting in India: An ecological analysis of new data from 112 districts. PLoS ONE.

[31] Kartini A., Subagio H.W., Hadisaputro S., Kartasurya M.I., Suhartono S., Budiyono B. (2019). Pesticide Exposure and Stunting among Children in Agricultural Areas. Int. J. Occup. Environ. Med..

[32] Coker E., Chevrier J., Rauch S., Bradman A., Obida M., Crause M., Bornman R., Eskenazi B. (2018). Association between prenatal exposure to multiple insecticides and child body weight and body composition in the VHEMBE South African birth cohort. Environ. Int..

[33] Kansiime W.K., Mugambe R.K., Atusingwize E., Wafula S.T., Nsereko V., Ssekamatte T., Nalugya A., Coker E.S., Ssempebwa J.C., Isunju J.B. (2022). Use of biomass fuels predicts indoor particulate matter and carbon monoxide concentrations; evidence from an informal urban settlement in Fort Portal city, Uganda. BMC Public Health.

[34] Amegah A.K., Quansah R., Jaakkola J.J.K. (2014). Household air pollution from solid fuel use and risk of adverse pregnancy outcomes: A systematic review and meta-analysis of the empirical evidence. PLoS ONE.

[35] Mishra V., Retherford R.D. (2007). Does biofuel smoke contribute to anaemia and stunting in early childhood?. Int. J. Epidemiol..

[36] Tielsch J.M., Katz J., Thulasiraj R.D., Coles C.L., Sheeladevi S., Yanik E.L., Rahmathullah L. (2009). Exposure to indoor biomass fuel and tobacco smoke and risk of adverse reproductive outcomes, mortality, respiratory morbidity and growth among newborn infants in south India. Int. J. Epidemiol..

[37] Kyu H.H., Georgiades K., Boyle M.H. (2009). Maternal smoking, biofuel smoke exposure and child height-for-age in seven developing countries. Int. J. Epidemiol..

[38] Amadu I., Seidu A.-A., Duku E., Boadu Frimpong J., Hagan Jnr J.E., Aboagye R.G., Ampah B., Adu C., Ahinkorah B.O. (2021). Risk factors associated with the coexistence of stunting, underweight, and wasting in children under 5 from 31 sub-Saharan African countries. BMJ Open.

[39] Brar S., Akseer N., Sall M., Conway K., Diouf I., Everett K., Islam M., Sène P.I.S., Tasic H., Wigle J. (2020). Drivers of stunting reduction in Senegal: A country case study. Am. J. Clin. Nutr..

[40] Keino S., Plasqui G., Ettyang G., van den Borne B. (2014). Determinants of stunting and overweight among young children and adolescents in sub-Saharan Africa. Food Nutr. Bull..

[41] Momberg D.J., Ngandu B.C., Voth-Gaeddert L.E., Cardoso Ribeiro K., May J., Norris S.A., Said-Mohamed R. (2021). Water, sanitation and hygiene (WASH) in sub-Saharan Africa and associations with undernutrition, and governance in children under five years of age: A systematic review. J. Dev. Orig. Health Dis..

[42] Mbuya M.N., Chidem M., Chasekwa B., Mishra V.K. (2010). Biological, social, and environmental determinants of low birth weight and stunting among infants and young children in Zimbabwe. Zimbabwe Working Papers.

[43] Rehfuess E., Mehta S., Prüss-Üstün A. (2006). Assessing Household Solid Fuel Use: Multiple Implications for the Millennium Development Goals. Environ. Health Perspect..

[44] Tishkoff S.A., Reed F.A., Friedlaender F.R., Ehret C., Ranciaro A., Froment A., Hirbo J.B., Awomoyi A.A., Bodo J.-M., Doumbo O. (2009). The genetic structure and history of Africans and African Americans. Science.

[45] Singh A., Ng’ang’a D., Gatari M.J., Kidane A.W., Alemu Z.A., Derrick N., Webster M.J., Bartington S.E., Thomas G.N., Avis W. (2021). Air quality assessment in three East African cities using calibrated low-cost sensors with a focus on road-based hotspots. Environ. Res. Commun..

[46] Linard C., Gilbert M., Snow R.W., Noor A.M., Tatem A.J. (2012). Population distribution, settlement patterns and accessibility across Africa in 2010. PLoS ONE.

[47] Singh A., Avis W.R., Pope F.D. (2020). Visibility as a proxy for air quality in East Africa. Environ. Res. Lett..

[48] WHO Regional Office for Africa Air Pollution. https://www.afro.who.int/health-topics/air-pollution.

[49] ICF Macro (2011). Demographic and Health Survey Supervisor’s and Editor’s Manual. MEASURE DHS Basic Documentation No. 4.

[50] Hammer M.S., van Donkelaar A., Li C., Lyapustin A., Sayer A.M., Hsu N.C., Levy R.C., Garay M.J., Kalashnikova O.V., Kahn R.A. (2020). Global Estimates and Long-Term Trends of Fine Particulate Matter Concentrations (1998–2018). Environ. Sci. Technol..

[51] van Donkelaar A., Hammer M.S., Bindle L., Brauer M., Brook J.R., Garay M.J., Hsu N.C., Kalashnikova O.V., Kahn R.A., Lee C. (2021). Monthly global estimates of fine particulate matter and their uncertainty. Environ. Sci. Technol..

[52] European Space Agency ESA/CCI Viewer. http://maps.elie.ucl.ac.be/CCI/viewer/download.php.

[53] Arino O., Gross D., Ranera F., Leroy M., Bicheron P., Brockman C., Defourny P., Vancutsem C., Achard F., Durieux L. GlobCover: ESA service for global land cover from MERIS. Proceedings of the 2007 IEEE International Geoscience and Remote Sensing Symposium.

[54] Li W., MacBean N., Ciais P., Defourny P., Lamarche C., Bontemps S., Houghton R.A., Peng S. (2018). Gross and net land cover changes in the main plant functional types derived from the annual ESA CCI land cover maps (1992–2015). Earth Syst. Sci. Data.

[55] Li W., Ciais P., MacBean N., Peng S., Defourny P., Bontemps S. (2016). Major forest changes and land cover transitions based on plant functional types derived from the ESA CCI Land Cover product. Int. J. Appl. Earth Obs. Geoinf..

[56] Grant R.L. (2014). Converting an odds ratio to a range of plausible relative risks for better communication of research findings. BMJ.

[57] Converting between Probabilities, Odds (Ratios), and Risk Ratios. https://cran.r-project.org/web/packages/effectsize/vignettes/convert_p_OR_RR.html.

[58] Flegal K.M. (2014). Bias in calculation of attributable fractions using relative risks from nonsmokers only. Epidemiology.

[59] WHO Ambient (Outdoor) Air Pollution. https://www.who.int/news-room/fact-sheets/detail/ambient-(outdoor)-air-quality-and-health.

[60] Chirande L., Charwe D., Mbwana H., Victor R., Kimboka S., Issaka A.I., Baines S.K., Dibley M.J., Agho K.E. (2015). Determinants of stunting and severe stunting among under-fives in Tanzania: Evidence from the 2010 cross-sectional household survey. BMC Pediatr..

[61] Yisak H., Gobena T., Mesfin F. (2015). Prevalence and risk factors for under nutrition among children under five at Haramaya district, Eastern Ethiopia. BMC Pediatr..

[62] Novignon J., Aboagye E., Agyemang O.S., Aryeetey G. (2015). Socioeconomic-related inequalities in child malnutrition: Evidence from the Ghana multiple indicator cluster survey. Health Econ. Rev..

[63] Leroy J.L., Habicht J.-P., de Cossío T.G., Ruel M.T. (2014). Maternal education mitigates the negative effects of higher income on the double burden of child stunting and maternal overweight in rural Mexico. J. Nutr..

[64] Krishna A., Mejía-Guevara I., McGovern M., Aguayo V.M., Subramanian S.V. (2018). Trends in inequalities in child stunting in South Asia. Matern. Child Nutr..

[65] Pillai V.K., Maleku A. (2019). Women’s education and child stunting reduction in India. J. Soc. Soc. Welf..

[66] Coker E.S., Amegah A.K., Mwebaze E., Ssematimba J., Bainomugisha E. (2021). A land use regression model using machine learning and locally developed low cost particulate matter sensors in Uganda. Environ. Res..

[67] World Bank GDP (Current US$)–Sub-Saharan Africa|Data. https://data.worldbank.org/indicator/NY.GDP.MKTP.CD?locations=ZG.

[68] Jorquera H., Montoya L.D., Rojas N.Y., Henríquez C., Romero H. (2019). Urban Air Pollution. Urban Climates in Latin America.

[69] Mauderly J.L., Burnett R.T., Castillejos M., Ozkaynak H., Samet J.M., Stieb D.M., Vedal S., Wyzga R.E. (2010). Is the air pollution health research community prepared to support a multipollutant air quality management framework?. Inhal. Toxicol..

[70] Jedrychowski W., Maugeri U., Jedrychowska-Bianchi I. (2002). Body growth rate in preadolescent children and outdoor air quality. Environ. Res..

