# Supplementary material for: Prenatal Exposure to Ambient PM2.5 and Early Childhood Growth Impairment Risk in East Africa
Source: Toxics. 2022 Nov 18;10(11):705. doi: 10.3390/toxics10110705 (PMC9699051; doi:10.3390/toxics10110705)
Supplement: Supplementary file 1 [file toxics-10-00705-s001.zip › toxics-2002734-supplementary.pdf]

toxics

Prenatal Exposure to Ambient PM2.5 and Early Childhood Growth Impairment Risk in East Africa

Kayan Clarke *, Adriana Rivas, Salvatore Milletich, Tara Sabo-Attwood and Eric S. Coker

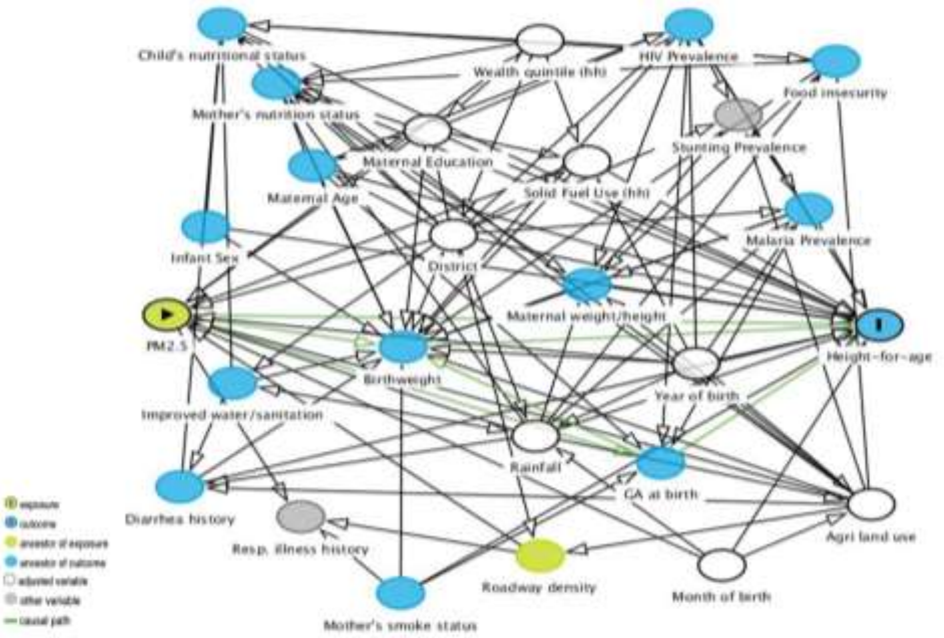

Figure S1. Directed Acyclic Graph showing the covariates that were selected as confounders because of their relationship with both the exposure (PM2.5) and the outcome (height-for-age).

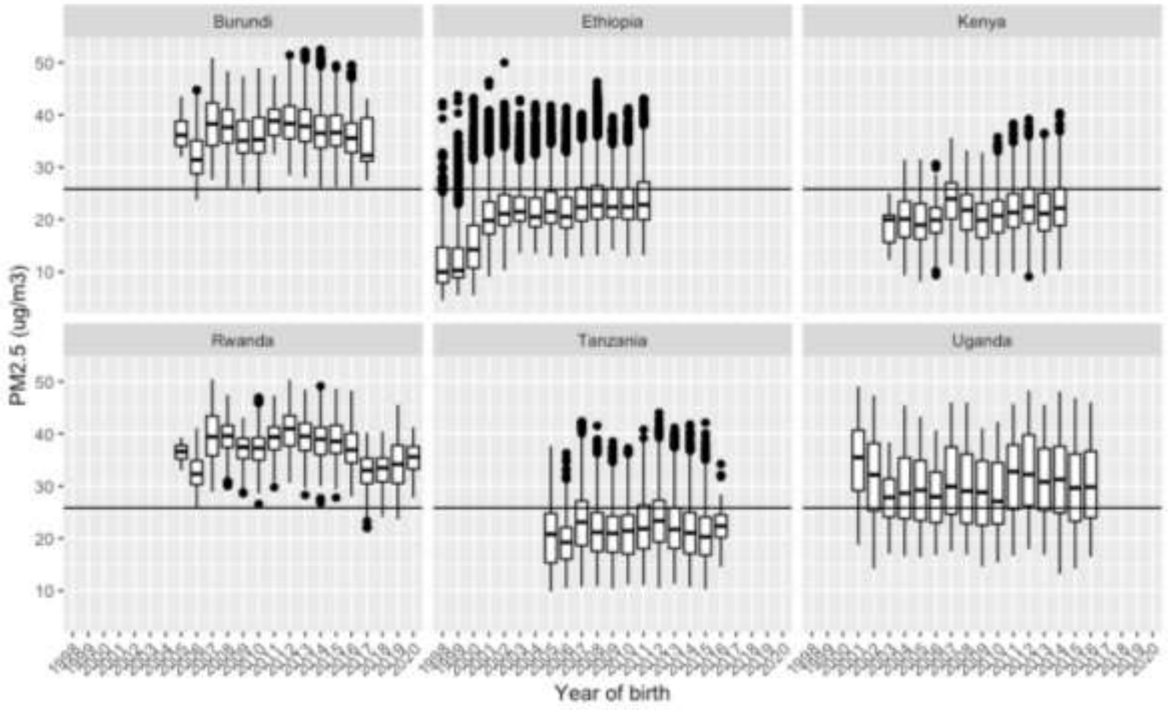

Figure S2. Long-term trend of prenatal PM2.5 exposures stratified by child's year of birth and by country. The horizontal black line shows the overall PM2.5 population average, (25.83 μg/m3), five times above the WHO annual recommended level at 5 μg/m^3.

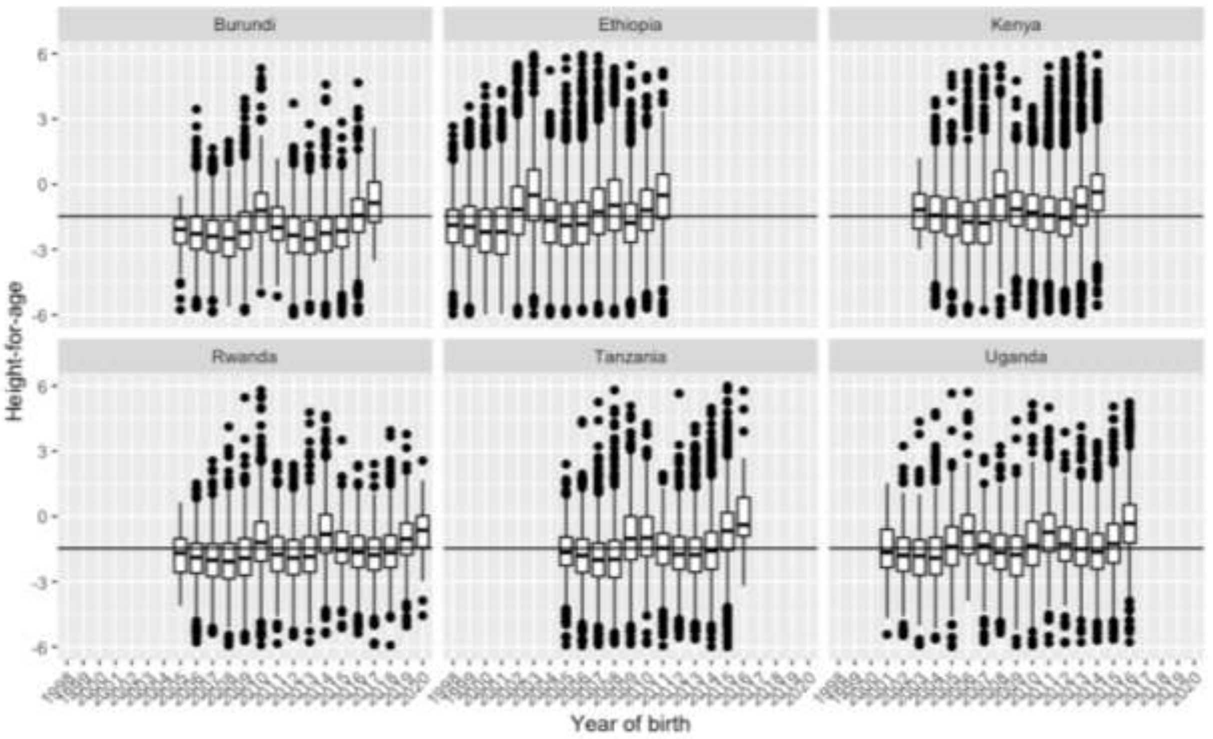

Figure S3. Long-term trend of height-for-age stratified by child's year of birth and by country. The horizontal black line shows the overall height standard deviation population average, -1.47 (95% CI: -1.48, -1.46).

Table S1. Crude and adjusted odds ratios for quartiles of prenatal PM2.5 exposure and stunting in children under 5 years old.

	Crude OR (95% CI)	aOR[a] (95% CI)	aOR[b] (95% CI)	aOR[c] (95% CI)
Prenatal PM2.5 exposure quartiles				
4.44–19.4 μg/m^3	reference			
19.4–24 μg/m^3	0.8785*** (0.8444, 0.9141)	1.0077 (0.965, 1.0525)	1.0089 (0.963, 1.0575)	0.990 (0.9422, 1.0411)
24–32.7 μg/m^3	0.981 (0.9432, 1.0204)	1.0419* (0.995, 1.0911)	1.0651** (1.0141, 1.1188)	1.0217 (0.960, 1.0873)
32.7–52.4 μg/m^3	1.4383*** (1.3839, 1.4947)	1.3185*** (1.2380, 1.4043)	1.3408*** (1.2565, 1.4307)	1.2560*** (1.1502, 1.3716)
Type of residence				
Urban		reference		
Rural		1.1095*** (1.0578, 1.1636)	1.1083*** (1.0522, 1.1673)	1.1144*** (1.0575, 1.1744)
Maternal education				
1st quartile		reference		
2nd quartile		0.959** (0.9256, 0.994)	0.9357*** (0.9015, 0.971)	0.9367*** (0.9022, 0.973)
3rd quartile		0.6825*** (0.6444, 0.7229)	0.6632*** (0.6238, 0.7052)	0.6624*** (0.6226, 0.7048)
4th quartile		0.4690*** (0.4148, 0.5303)	0.4518*** (0.3946, 0.5173)	0.4399*** (0.3834, 0.504)
Wealth index				
1st quintile		reference		
2nd quintile		0.9171*** (0.8800, 0.956)	0.9239*** (0.8845, 0.965)	0.9134*** (0.8740, 0.955)
3rd quintile		0.8059*** (0.7715, 0.8418)	0.8222*** (0.7855, 0.8607)	0.8147*** (0.7779, 0.8532)
4th quintile		0.6580*** (0.6281, 0.6894)	0.6702*** (0.6385, 0.7035)	0.6617*** (0.6300, 0.6950)
5th quintile		0.4334*** (0.4073, 0.4611)	0.4383*** (0.4107, 0.4676)	0.4324*** (0.4050, 0.4616)
Use of polluting fuel				
No		reference		
Yes		1.1832** (1.0245, 1.3665)	1.1452* (0.980, 1.3383)	1.1534* (0.986, 1.3499)
Breastfed				
No			reference	
Yes			0.8500*** (0.7546, 0.958)	0.8494*** (0.7533, 0.958)
Postnatal PM2.5 exposure				1.0061*** (1.0018, 1.0104)

Logistic regression where the covariates are the estimated odds ratio of the outcome. ***$p < 0.01$, **$p < 0.05$, *$p < 0.1$
[a]Model 1: Smoothing terms for year of birth, percent crop and random effects for primary sampling unit, mother, month of birth, and country. [b]Model 2: Ever breastfed added to the model. [c]Model 3: Postnatal PM2.5 exposure added to the model. CI: Confidence Interval; OR: Odds Ratio; aOR: adjusted Odds Ratio

Table S1

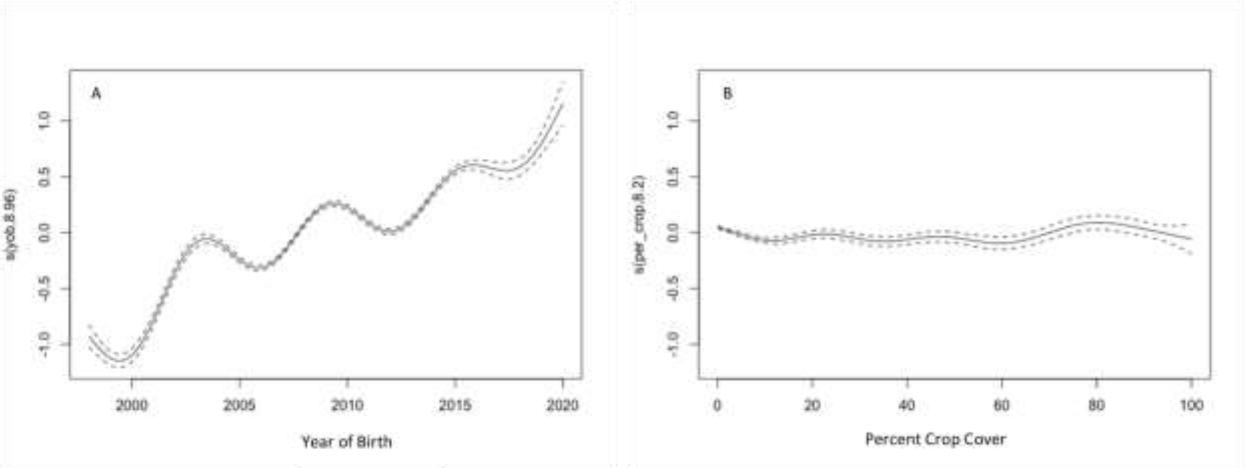

Figure S4. Plots of non-linear effects on height-for-age for (A) year of birth and (B) percent crop cover.

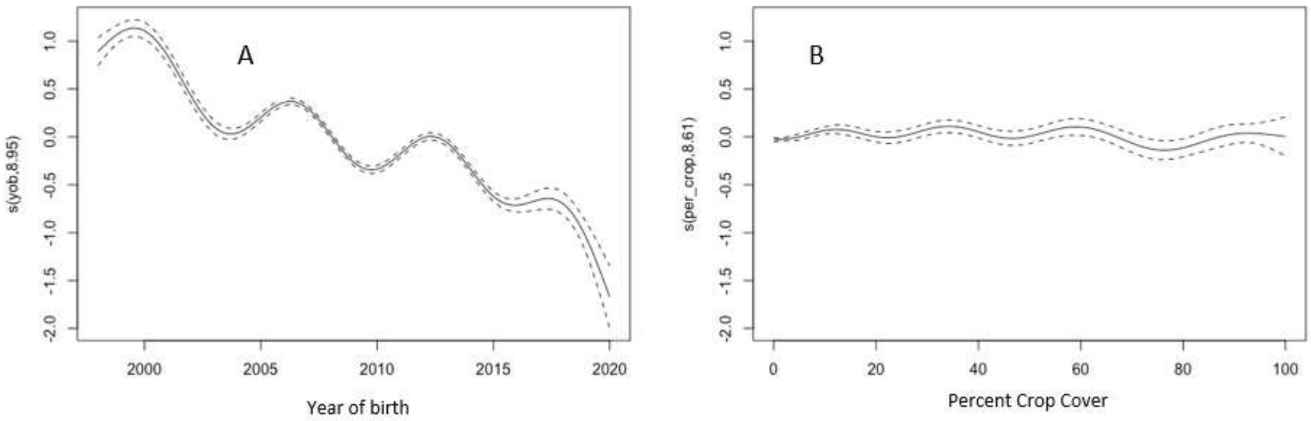

Figure S5. Plots of non-linear effects on stunitng for (A) year of birth and (B) percent crop cover.
